# Chronic Bilateral Olecranon Bursitis: A Case Report

**DOI:** 10.7759/cureus.65881

**Published:** 2024-07-31

**Authors:** Ahmad Y Nassar, Bashour Hanna, Youssef Abou Chahine, Mahmoud Ayche, Ahmad Srour

**Affiliations:** 1 Orthopedics, Paris Shoulder Unit, Clinique Bizet, Paris, FRA; 2 Orthopedics, Hôpital de Voiron, CHU Grenoble Alpes, Voiron, FRA; 3 Orthopedics, Mubadala Health, Dubai, ARE; 4 Orthopedics, Arras Hospital, Arras, FRA

**Keywords:** chronic, bursitis, elbow swelling, elbow joint, olecranon

## Abstract

Olecranon bursitis is a common condition that primarily affects men between the ages of 30 and 60. Although the conservative treatment of acutely inflamed olecranon bursitis is relatively straightforward, managing chronic olecranon bursitis can be challenging. In this publication, we report a case of rare bilateral chronic olecranon bursitis and discuss the rationale for choosing the best treatment option.

## Introduction

Olecranon bursitis is a condition characterized by inflammation of the olecranon bursa, often resulting from microtrauma [1]. This condition accounts for 0.01-0.1% of hospital admissions and is more prevalent in males aged 30 to 60 [2]. Olecranon bursitis can be classified as acute or chronic and can also be either septic or non-septic [3]. Chronic bilateral olecranon bursitis is a rare but debilitating condition, especially for individuals engaged in repetitive manual labor, such as truck drivers.

## Case presentation

A 73-year-old male truck driver presented to an outpatient clinic with a several-year history of progressively increasing bilateral elbow swelling. He reported no skin pain or pruritus and denied having fever, chills, weight loss, or gastrointestinal or respiratory symptoms. There was no notable travel history. He had a medical history of asthma and cirrhosis of the liver.

**Figure 1:**
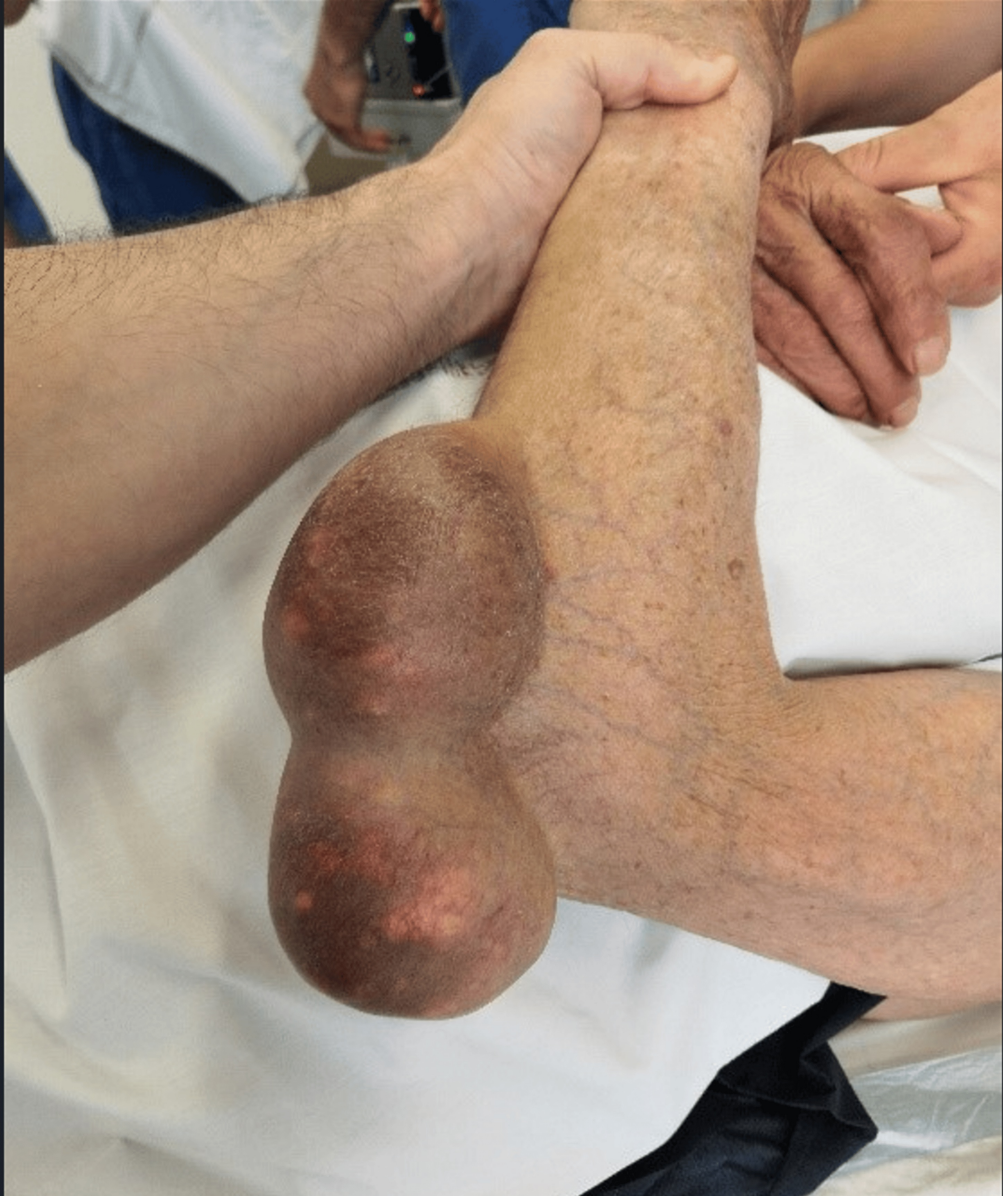
Left elbow with an olecranon bursitis mass

**Figure 2:**
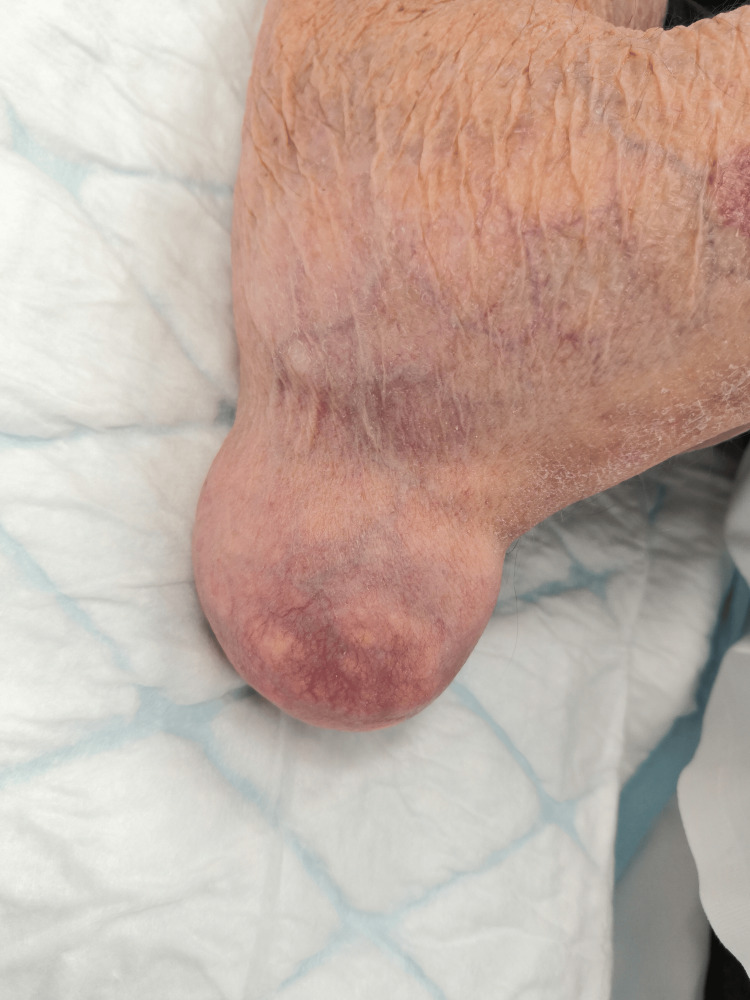
Right elbow with an olecranon bursitis mass

Physical examination revealed significant swelling overlying the olecranon processes of both elbows. The swellings were spherical, firm in consistency, and non-tender to palpation (Figures 1, 2). The patient reported frequent and repetitive pressure on both elbows while working. The swelling worsened after a long day of driving, especially if he had been forced to lean on his elbows for extended periods. Blood tests were within normal limits.

**Figure 3:**
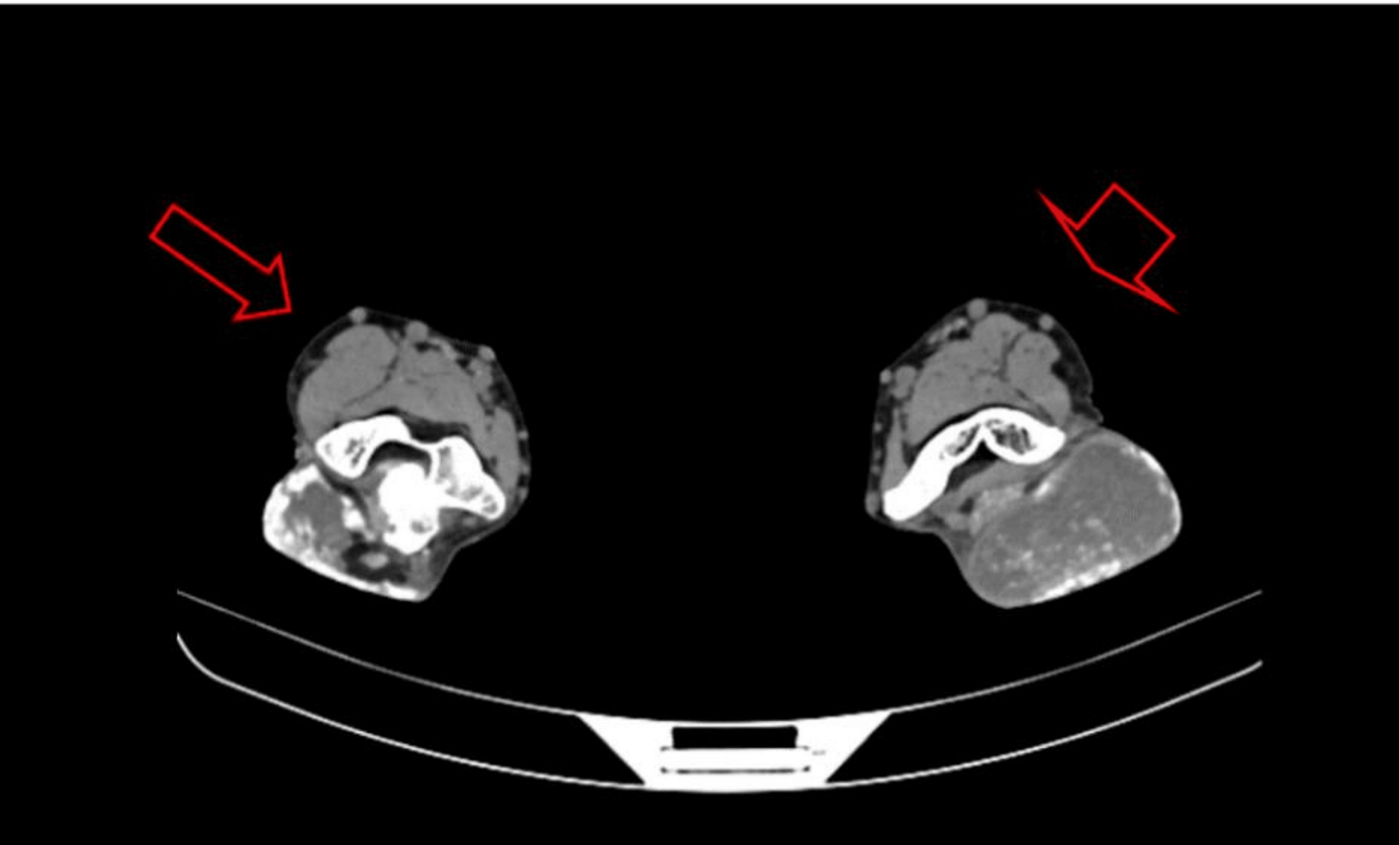
CT scan axial view of both elbows showing bilateral soft tissue fluid-filled cystic masses

**Figure 4:**
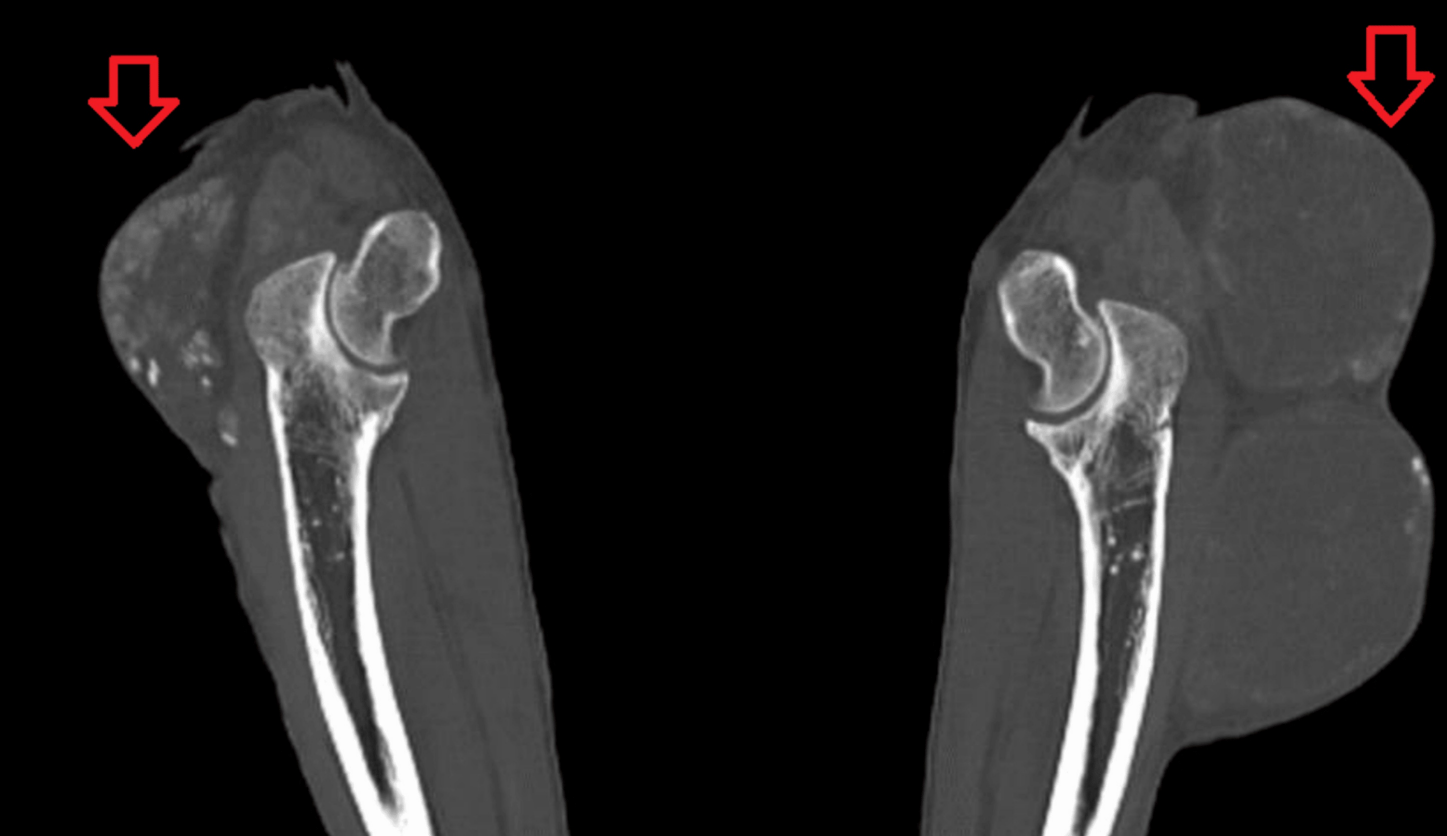
CT scan sagittal view of both elbows showing bilateral soft tissue fluid-filled cystic masses

CT scan imaging of both elbows revealed bilateral soft tissue fluid-filled cystic masses consistent with chronically inflamed olecranon bursae. The left elbow bilobular mass measured 7.7 x 4.8 cm for the upper lobe and 8.4 x 7.5 x 5.5 cm for the lower lobe, while the right elbow mass measured 6.6 x 6.0 x 3.2 cm (Figures 3, 4).

Multiple trials of conservative treatments, including rest, regular icing of the affected areas, and analgesics as needed, failed to treat the inflamed bursae. Since the patient is a truck driver and the bursitis began to impact his work performance, surgical resection of the inflamed bursae was planned.

**Figure 5:**
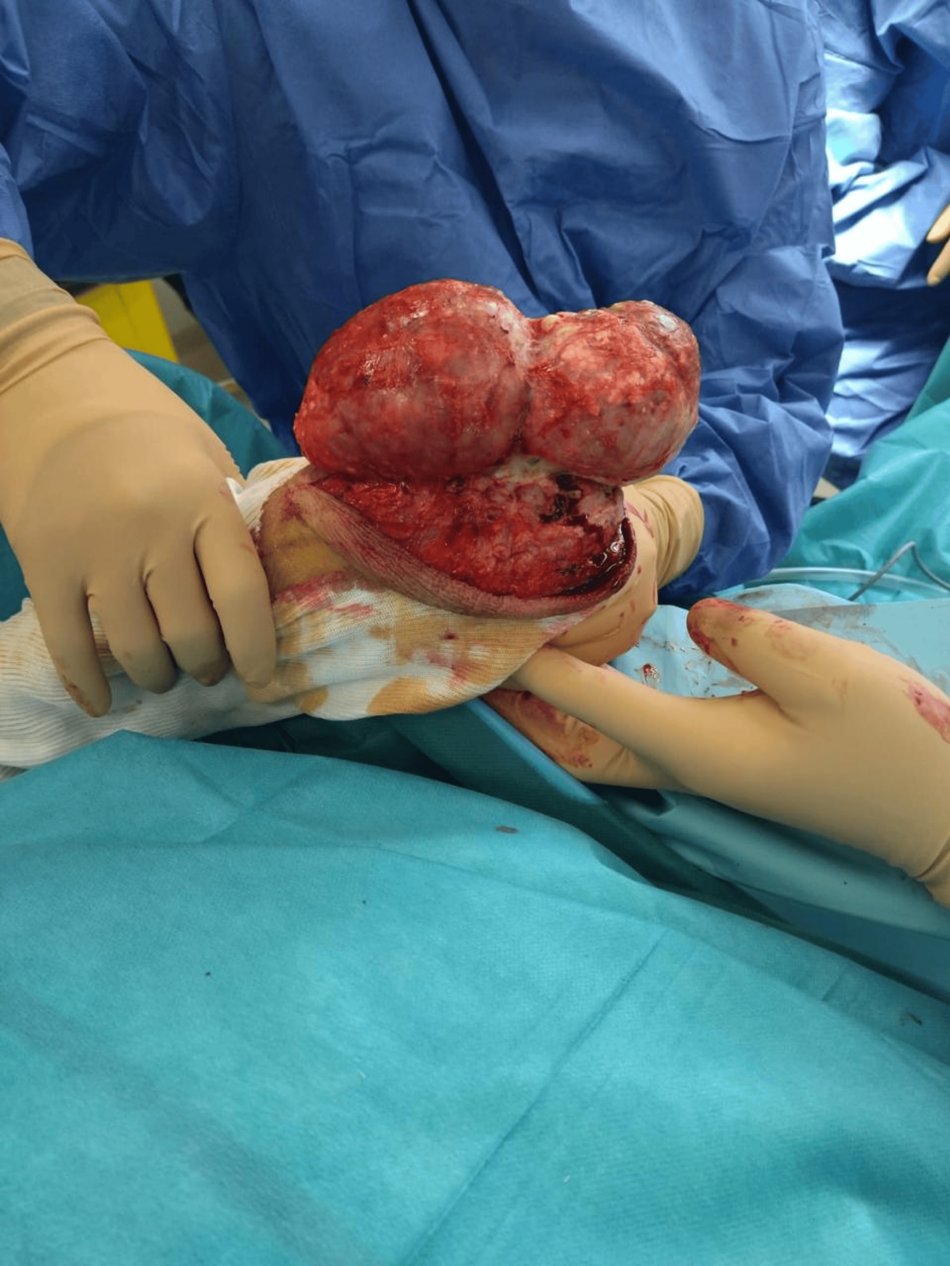
Exploration of the left elbow olecranon bursitis mass

**Figure 6:**
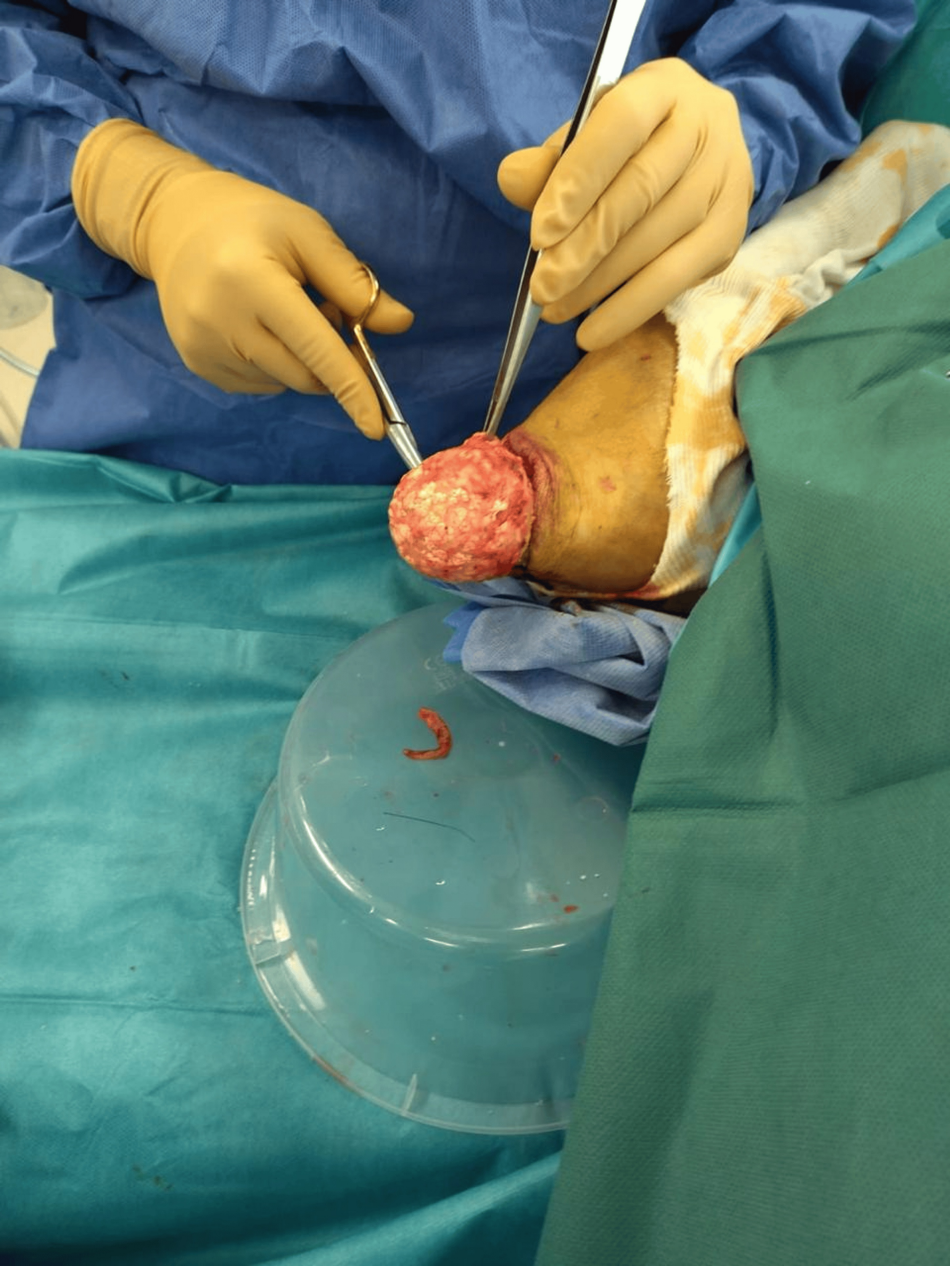
Exploration of the right elbow olecranon bursitis mass

The patient was hospitalized, and surgical excision of both olecranon bursae was performed in the operating room under general anesthesia. The patient was positioned in the supine position, and an incision of the skin just above the bursae was made, followed by dissection of the subcutaneous tissues and removal of the fibrous and inflammatory tissues around the inflamed bursae (Figures 5, 6).

**Figure 7:**
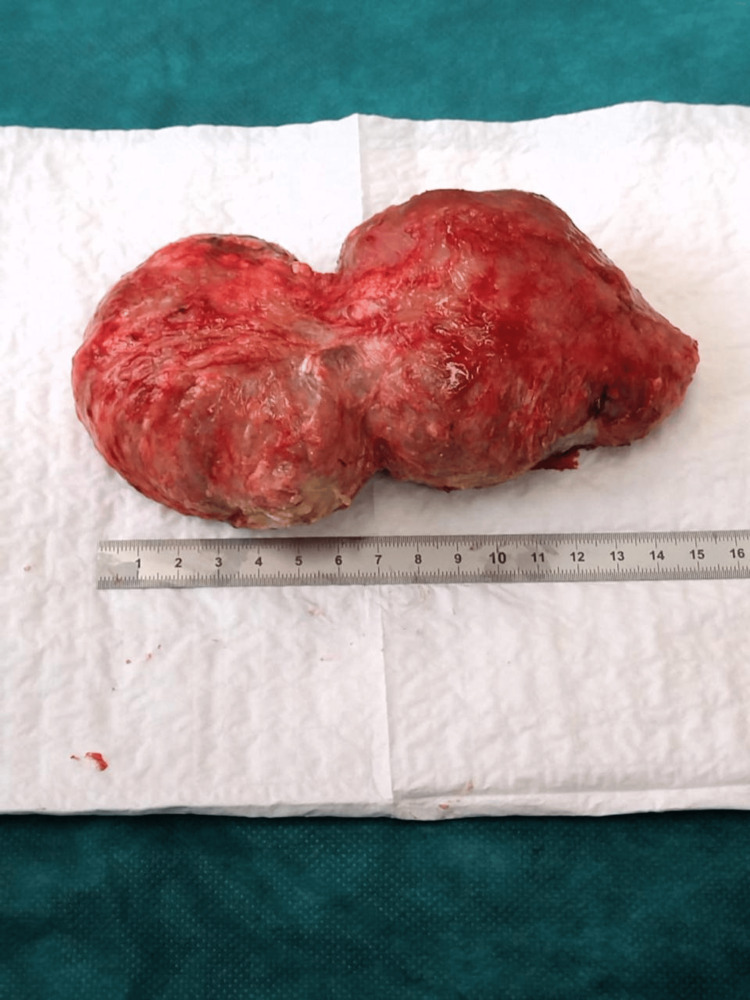
15 cm measurement of the left elbow olecranon bursitis mass

**Figure 8:**
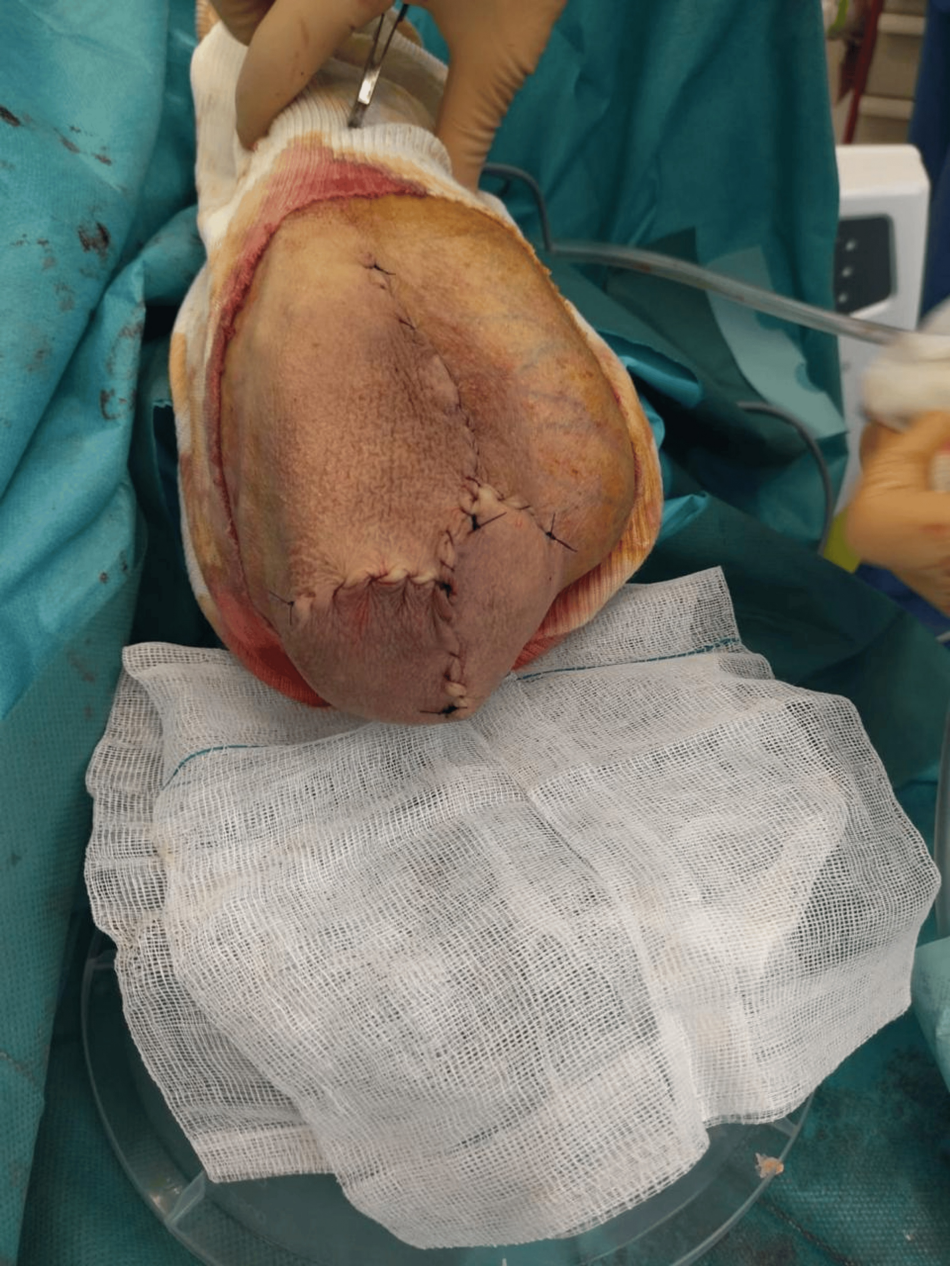
Left elbow post removal of the chronic bursitis mass

The masses were resected bilaterally and sent to the pathology laboratory (Figure 7). Hemostasis was achieved using electrocautery, and the skin was closed with non-absorbable suture material in a continuous fashion (Figure 8). Histopathologic examination of both elbows revealed chronic fibro-inflammatory changes of the resorptive granulomatous type, with no signs of malignancy or sepsis, consistent with chronic bilateral bursitis.

The patient was then advised to follow a home exercise program to strengthen the muscles around the affected joints, reducing the risk of recurrence.

Follow-up over the next six months was uneventful, except for bilateral collections at the operation site on day 14 post-op. These collections were drained in the operating room under general anesthesia and were found to be hematomas. No postoperative complications occurred. The patient reported a full range of motion in both elbows with no signs of recurrent bursitis.

## Discussion

Chronic bilateral olecranon bursitis is a rare condition with significant implications for patients engaged in repetitive manual labor, such as truck drivers. Treatments vary from conservative approaches like rest, ice, and analgesics to more invasive procedures like aspiration or surgical removal [4]. For chronic olecranon bursitis, symptomatic treatment involving elevation, splinting, ice, and anti-inflammatories is the preferred initial approach [5,6]. While aspiration and steroid injection into non-infected bursitis have been attempted to shorten the disease's course, some studies have challenged this approach, citing a 10% contamination risk leading to infection [7].

Surgical excision has been confirmed as an effective treatment for chronic olecranon bursitis unresponsive to conservative methods [5]. Other reasons for surgical removal include septic bursitis [8-12] and the presence of an olecranon spur [13].

In our practice, we emphasize the importance of rehabilitating the affected joints to prevent recurrence. A well-designed exercise regimen that includes regular stretching and strengthening exercises can be instrumental in avoiding further complications.

## Conclusions

Treating chronic bilateral olecranon bursitis presents numerous challenges and varies depending on the specific circumstances. While conservative management is typically the first-line treatment, aspiration or surgical excision may be necessary in some cases. Rehabilitation of the affected joints is crucial for preventing recurrence.
